# Understanding the Molecular Mechanism of Thermal and LA-Catalysed Diels–Alder Reactions between Cyclopentadiene and Isopropyl 3-Nitroprop-2-Enate

**DOI:** 10.3390/molecules28145289

**Published:** 2023-07-08

**Authors:** Ewa Dresler, Aneta Wróblewska, Radomir Jasiński

**Affiliations:** 1 Łukasiewicz Research Network—Institute of Heavy Organic Synthesis “Blachownia”, Energetyków 9, 47-225 Kędzierzyn-Koźle, Poland; ewa.dresler@icso.lukasiewicz.gov.pl; 2 Centre of Molecular and Macromolecular Studies, Polish Academy of Sciences, Sienkiewicza 112, 90-363 Lodz, Poland; aneta.wroblewska@chemia.uni.lodz.pl; 3 Institute of Organic Chemistry and Technology, Cracow University of Technology, Warszawska 24, 31-155 Cracow, Poland

**Keywords:** Diels–Alder reaction, nitroalkene, molecular mechanism, molecular electron density theory, DFT calculations

## Abstract

The molecular mechanism of the Diels–Alder reaction with the participation of cyclopentadiene and isopropyl 3-nitroprop-2-enate was examined based on wb97xd/6-311+G(d) (PCM) quantum chemical calculations. It was found that the type of mechanism for the conversion of addends depends significantly on the reaction conditions. In less-polar environments, a one-step polar mechanism is realised. In more polar solvents, the formation of “extended”-type zwitterionic intermediates is possible. In contrast, in the presence of an LA-type catalyst, the one-step mechanisms are replaced by respective stepwise mechanisms with zwitterionic or heterocyclic intermediates.

## 1. Introduction

The Diels–Alder (DA) reaction with the participation of cyclopentadiene **1** is a most universal strategy for the preparation of different types of compounds containing norbornene carbon skeleton [1,2]. The potential of structures obtained in this way is especially greater when the norbornene molecular segment is conjugated with the nitro group. This stimulates the possibility of further functionalisation for amines [3], nitrile N-oxides [4,5], oximes [6], nitronates [7,8] and many others [9,10]. Additionally, the nitro group’s presence within organic molecules stimulates its bioactive function [11,12,13]. The introduction of a NO_2_ group to the norbornene skeleton is possible via direct nitration or the substitution of halogen atoms [14,15]. However, the easiest strategy for the preparation of nitronorbornenes is based on DA reactions with the participation of conjugated nitroalkenes. These processes have recently been a special subject of our comprehensive research [16,17,18,19].

**Scheme 1:**
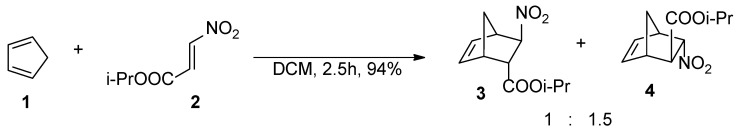
The observed experimental course of the DA reaction between cyclopentadiene **1** and isopropyl 3-nitroprop-2-enate **2** leading to stereoisomeric nitronorbornenes **3** and **4**.

**Scheme 2:**
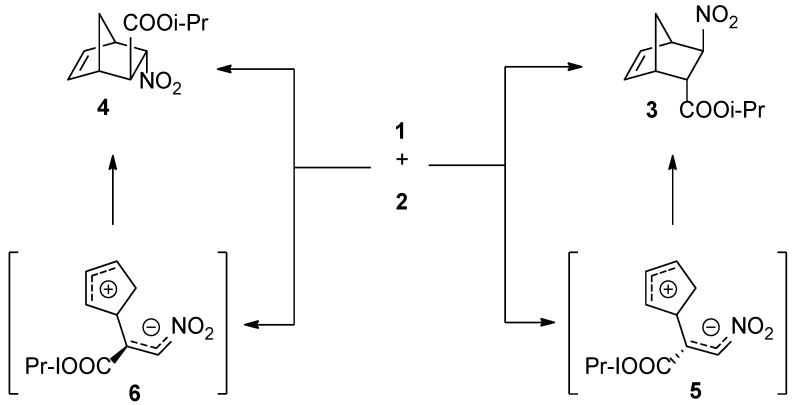
Theoretically possible one-step and stepwise formation of DA adducts in the reactions between cyclopentadiene **1** and isopropyl 3-nitroprop-2-enate **2**.

Some time ago, Corey and coworkers [20] conducted DA reactions of cyclopentadiene 1 with isopropyl 3-nitroprop-2-enate 2. It was found that in the dichloromethane (DCM) solution, the mentioned process led to endo-nitronorbornene 4 as the major product (Scheme 1). Additionally, *exo*-nitronorbornene 3 was formed as the minor adduct. The mechanism of this reaction was, however, unclear (Scheme 2). Due to the electrophilic nature of conjugated nitroalkenes [21,22], in this case, the “classical” one-step mechanism can compete with the stepwise mechanism realised via the zwitterionic intermediate (5 and/or 6). Interesting examples of these types of DA reactions were recently detected [23,24,25]. Additionally, recent publications suggest the possibility of the formation of a DA-type adduct via stepwise sequence via the Hetero Diels–Alder stage and further [3.3]-sigmatropic rearrangement of primary formed internal nitronate [16,18]. This is possible inter alia in the case of DA reactions with the participation of trifluoromethylated nitroalkenes or 1-cyano-substituted nitroethenes. As a consequence, mechanistic aspects of the title reaction require deeper exploration. For this purpose, we used data derived from the calculations based on the density functional theory (DFT). In this computational study, we simulated the presence of DCM (tested experimentally) and more polar nitromethane. Lastly, we also examined the potential catalytic effect derived from the presence of a Lewis acid (LA) catalyst.

## 2. Results and Discussion

Our research started with the exploration of mechanistic aspects of the title reaction in the DCM solution. The results of the wb97xd/6-311+G(d) (PCM—Polarisable Continuum Model) calculations suggest that the qualitative description of enthalpy profiles for both considered cycloaddition paths were similar. In particular, in both cases, the same type of critical structure was localised between the valley of reagents and the valley of adducts: molecular pre-reaction complex (MC) and the single transition state (TS).

**Figure 1:**
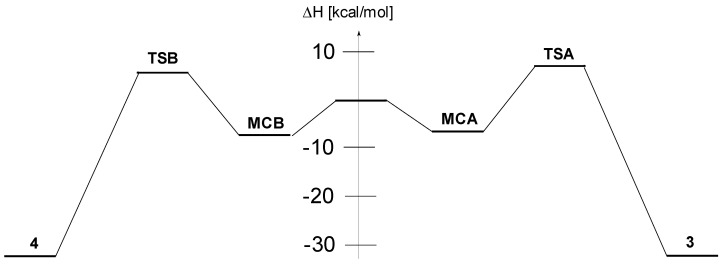
Enthalpy profiles for the reactions between cyclopentadiene **1** and isopropyl 3-nitroprop-2-enate **2** in the DCM environment in light of the wb97xd/6-311+G(d) (PCM) calculations.

**Figure 2:**
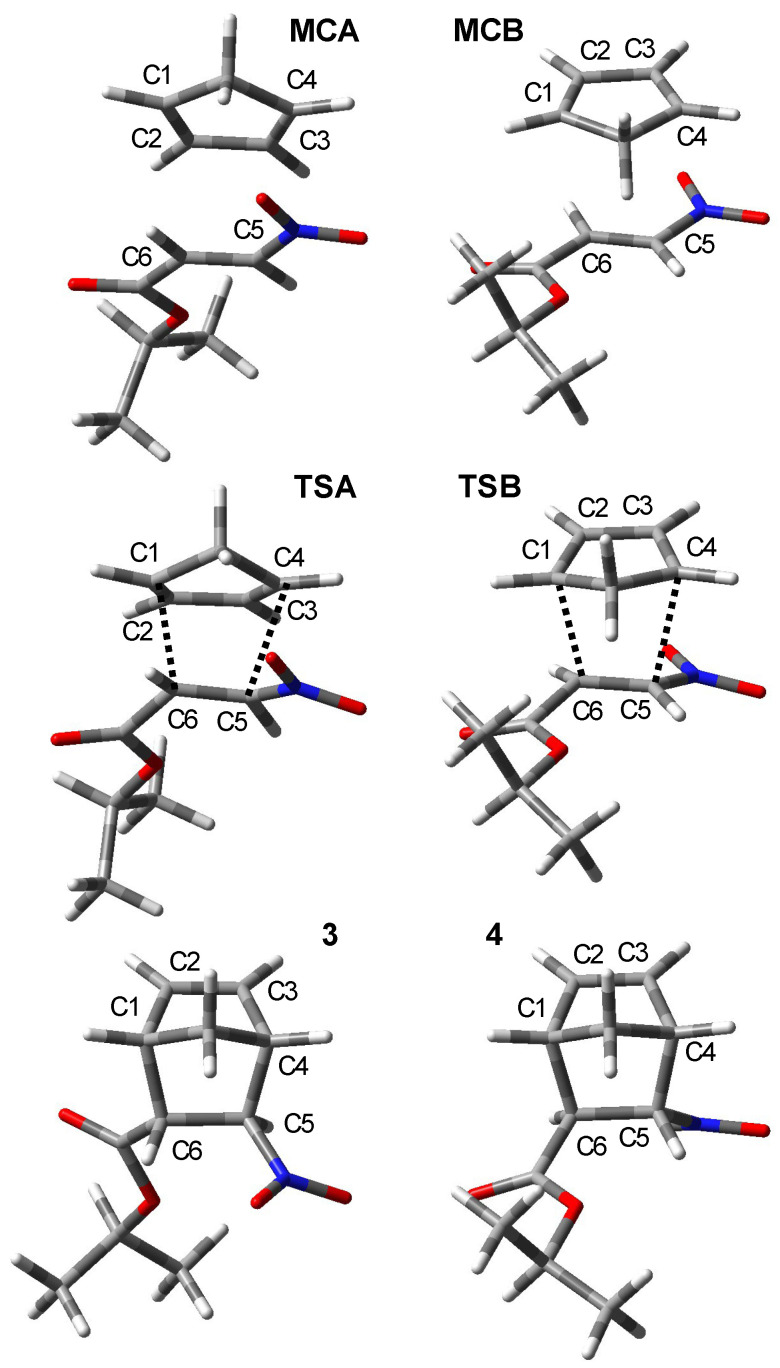
Views of critical structures of the reactions between cyclopentadiene **1** and isopropyl 3-nitroprop-2-enate **2** in the DCM environment in light of the wb97xd/6-311+G(d) (PCM) calculations.

The interactions between addend molecules led, at the first reaction stage, to forming the molecular pre-reaction complex (MCA and MCB, respectively, for paths A and B). The decrease in the enthalpy of the reaction system of about 6 kcal/mol was a consequence of this transformation (Table 1 and Table 2, Figure 1). It should be mentioned that the substantial reduction in the entropy was realised at the same time. Therefore, Gibbs free energy (ΔG) of the formation of MCs took a positive value, which excluded the possibility of MCs as stable intermediates. Therefore, valleys of MCs on the reaction profile can be considered to exist at the enthalpy, but not at the Gibbs energy surface. From a structural point of view, MCs should be treated as a molecular pair stabilised via coulombic interactions. Subsequently, no new sigma-bonds were formed at this stage (Table 3, Figure 2). Additionally, no electron density flux between substructures was observed at this stage (global electron density transfer GEDT = 0.00). The substructures of addents adopted orientations which were favourable for the further formation of the transition state. Similar pre-reaction complexes were recently detected regarding different-type intermolecular cycloaddition reactions [26,27,28].

The further reduction of key interatomic distances along the reaction coordinates led to the formation of transition states (TSA and TSB, respectively, for paths A and B). This was accompanied by an increase in the enthalpy of the reaction system by 6.9 kcal/mol and 5.9 kcal/mol regarding paths A and B, respectively. Including the entropy factors in the considerations shows that activation barriers ΔG on considered paths were equal to 22.1 kcal/mol and 21.1 kcal/mol, respectively. Therefore, the cycloaddition channel was favoured with the endo-orientation of the nitro group within the formed adduct. The competitive channel leading to the *exo*-nitro cycloadduct cannot be treated as forbidden from the kinetic point of view. Therefore, the obtained values correlated well with the stereoselectivity experimentally observed.

Within both TSs, the key interatomic distances were substantially reduced. It is interesting that the distance C6–C1 was evidently shorter than the second one (C4–C5). This was not a consequence of the sterical effects [29] and can be easily explained based on the analysis of the local electrophilicities and nucleophilicities of addents [30]. It was found that within the considered reagents pair, the cyclopentadiene 1 played the role of nucleophilic agent (ω = 0.83 eV, N = 3.36 eV), whereas the nitroalkene must be treated as an electrophile (ω = 2.31 eV). Next, the most nucleophilic centre was located at the C1 carbon atom of cyclopentadiene, whereas the most electrophilic centre was located at the C6 carbon atom of the nitrovinyl moiety. Therefore, the reaction proceeded under the control of the interactions between the most activated positions at the unsaturated moieties of addends.

Analysis of the electron density distribution within TSs exhibits that the electron density transfer from the substructure of cyclopentadiene to the substructure of nitroalkene was observed in both cases. Therefore, formally, both cycloaddition channels should be treated as Forward Electron Density Flux (FEDF) [29] processes. The internal reaction coordinate (IRC) calculations connected directly localised TSs within valleys of respective MCs and respective products. All attempts for the localisation of alternative paths leading to adducts via hypothetical zwitterionic intermediates were not-successful. Therefore, the analysed reaction can be treated as a polar but one-step Diels–Alder reaction [31].

**Figure 3:**
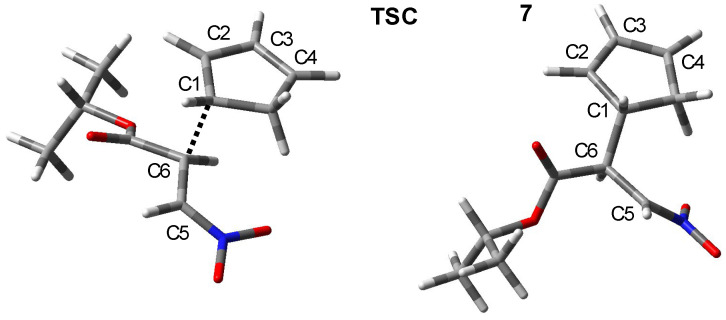
Views of **TSC** and zwitterion **7** structures of the reactions between cyclopentadiene **1** and isopropyl 3-nitroprop-2-enate **2** in the nitromethane environment in light of the wb97xd/6-311+G(d) (PCM) calculations.

We performed a similar study for the analogous reaction in a more polar solvent (nitromethane). It was found that the energy profiles of both considered cycloaddition channels were similar, as observed in the DCM solution. Next, the quantitative descriptions of these profiles were in the nitromethane, almost identical to the DCM. The structural characteristics of TSA and TSB were also similar in both considered solvents. Unexpectedly, however, the additional path C of the consumption of substrates was detected in the nitromethane solution. This was the reaction channel leading to the zwitterion 7. The first stage of this path was the formation of MCA, which can be considered a common intermediate for paths A and C. This was confirmed via IRC analysis. The further conversion of MCA can lead to the transition state TSC. This TS reduced the key interatomic distance C6-C1 to 1.9 Å. In contrast, the C4-C5 interatomic distance was beyond the range typical for C-C bonds within transition states [32,33,34]. TSC (Figure 3) subsequently exhibited an evidently more polar nature than TSA and TSB. It should be noted that the path C should be treated as forbidden from the kinetic point of view because the activation energy was more than 12 kcal/mol higher, as in the case of paths A and B (ΔH = 18.9 kcal/mol, ΔG = 33.1 kcal/mol). The further reduction in the C6–C1 distance led to the formation of a zwitterion 7 molecule. Its zwitterionic nature was clearly confirmed by the value of GEDT (Table 3). It is important that due to the Z-type, “extended” conformation [35], the direct cyclisation of 7 into cycloadduct was impossible. Its cyclisation proceeded via the stage of the dissociation into individual reagents and further conversion via A or B cycloaddition paths.

The introduction of BF_3_ as the LA-type catalyst to the reaction environment enforced fundamental changes in molecular mechanisms on both considered cycloaddition paths. The BF_3_/nitroalkene molecular complex ([**2**/BF_3_]) was formed within the first reaction stage. This was realised without any activation barrier and was accompanied by a reduction in the enthalpy of the reaction system of about 6 kcal/mol. Within this complex, the boron atom was located near the oxygen atom of the nitroalkene molecular segment. Similar complexes between the nitro group and Lewis Acids were detected recently [36,37,38]. This intermediate was stabilised via coulombic interactions between the boron shell, characterised by a positive partial charge, and the nitro group’s oxygen atom, characterised by a negative partial charge. Further chemical transformations were realised via the interaction of this complex with the cyclopentadiene 1 molecule according to multi-step mechanisms. These transformations exhibited, however, completely different natures depending on the considered reaction path.

**Figure 4:**
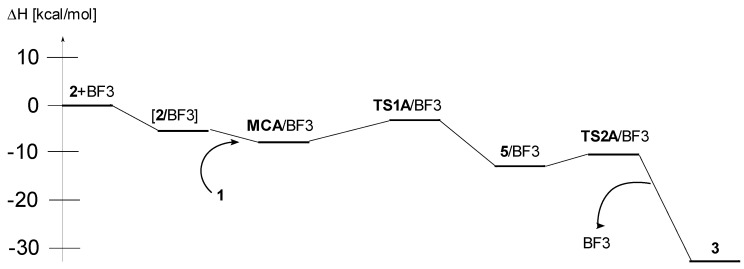
Enthalpy profiles for the BF_3_-catalysed **1** + **2** → **3** reaction in the DCM environment in light of the wb97xd/6-311+G(d) (PCM) calculations.

The enthalpy profile of the reaction finally leading to the *exo*-nitronorbornene 3 is presented in Figure 4. Within this profile, four critical points were localised between an area of the starting molecular system (individual **1** and [**2**/BF_3_]) and an area of the final product.

**Figure 5:**
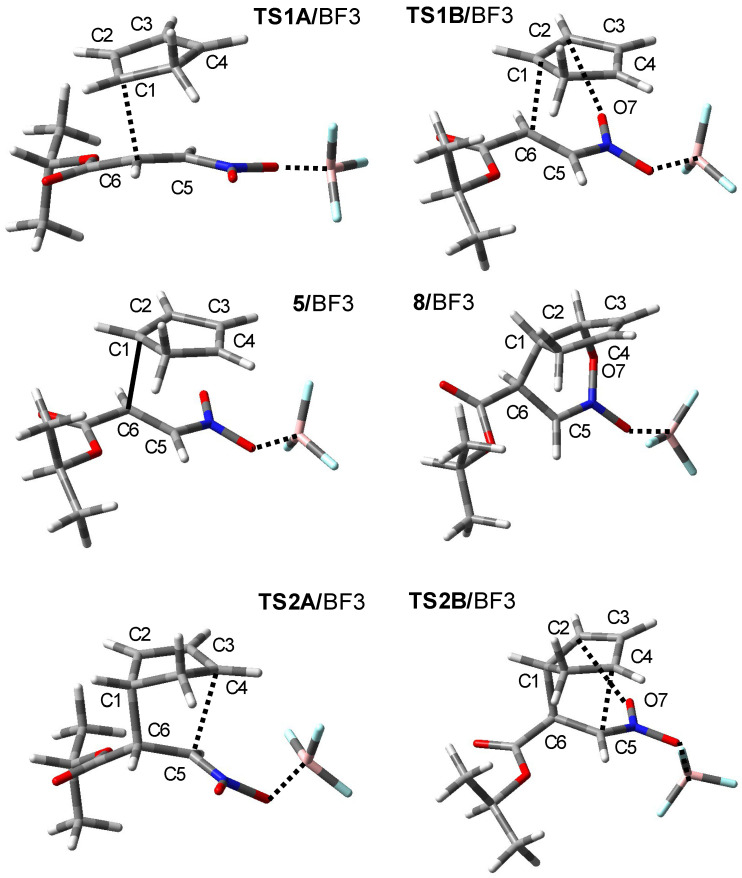
Views of critical structures of the BF_3_-catalysed reactions between cyclopentadiene **1** and isopropyl 3-nitroprop-2-enate **2** in the DCM environment in light of the wb97xd/6-311+G(d) (PCM) calculations.

The first reaction step was the formation of the molecular complex MCA/BF_3_. This step was realised without the activation barrier. The enthalpy of the reaction system was reduced at this stage by 7.7 kcal/mol. Within the MCA/BF_3_, no new bonds were formed (Figure 5). The further movement of the reaction system via the reaction coordinates led to the area of the transition state TS1A/BF_3_. This was accompanied by an increase in the enthalpy of the reaction system by about a few kcal/mol. Including the entropy factors in the considerations shows that activation barriers ΔG on the considered path equalled 12.4 kcal/mol. Within this structure, the distance between reaction centres C1 and C6 was reduced up to about 2.2 Å. On the other hand, the interatomic distance C4–C5 existed beyond the area typical for new C-C bonds within transition states [32]. Subsequently, the electron density transfer from the substructure of cyclopentadiene to the nitrovinyl segment was observed. Therefore, according to Domingo’s terminology [31], this TS can be classified as polar. The reduction of the C1–C6 distance directed the reaction system into an area of the complex of intermediate **5** with BF_3_. Within this molecule, the key C1–C6 bond exhibited a length of about 1.58 Å. Next, the analysed intermediate was characterised by a strongly zwitterionic nature and “cyclic” conformation for the contrast of the zwitterion **7** mentioned above. This was confirmed by the GEDT value, which was equal to almost 1e (Table 3). The zwitterion **5** was unstable from the thermodynamic point of view and easy to convert to the target norbornene system **3**. This process was realised via the TS1A/BF_3_ transition state. Within this TS, the C4–C5 interatomic distance was substantially reduced up to 2.3 Å. The IRC calculation connected this structure clearly with the valleys of intermediate and product **3**. Therefore, for the contrast of the non-catalysed cycloaddition, a BF_3_-promoted reaction **1** + **2** → **4** was realised according to the stepwise mechanism with the zwitterionic intermediate.

**Figure 6:**
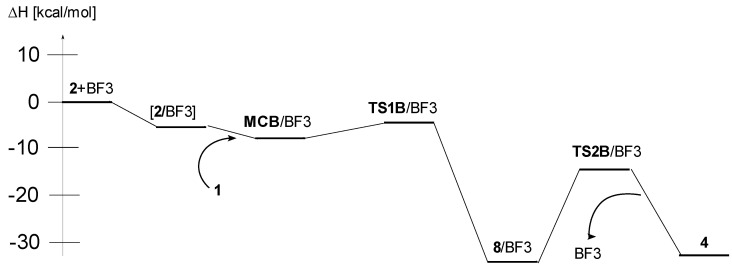
Enthalpy profiles for the BF_3_-catalysed **1** + **2** → **4** reaction in the DCM environment in light of the wb97xd/6-311+G(d) (PCM) calculations.

Alternatively, the molecular complex [2/BF_3_]) can react with the cyclopentadiene **1** on the competitive channel, finally leading to *endo*-nitronorbornene 4. This reaction proceeded via four critical points (Figure 6). Similarly, as in the case of path A, discussed above, the first step was connected with the barrier-less formation of the respective pre-reaction complex within this reaction way. This is an MCB/BF_3_ complex. It is a “meeting complex” with a sandwich structure that does not show the nature of a charge-transfer complex (GEDT = 0.00 e). The consequence of the MCB/BF_3_ complex formation was a reduction in the enthalpy of the reaction system by about a few kcal/mol.

The further conversion of the molecular MCB/BF_3_ complex was its transformation to the TS1B/BF_3_ transition state. This TS reduced the distance between reactions C1 and C6 to about 2.2 Å. Unexpectedly, at the same time, the second interatomic distance C2–O7 (Figure 5) was also substantially shortened instead of the expected distance C4–C5. Formally, the considered TS was evidently like transition states observed within the transition states of Hetero Diels–Alder (HDA) reactions [33,34]. This was confirmed via IRC calculations, which connected TS1B/BF_3_ with the 1,2-oxazine N-oxide 8 structure linked with BF_3_ (8/BF_3_) instead of the expected *endo*-nitronorbornene 4 linked by BF_3_. The rearrangement of the 1,2-oxazine N-oxide 8 into the target norbornene structure was possible via the second transition state (TS1B/BF_3_). Within this TS, the C1–O6 distance was practically unchanged. Subsequently, the C4–C5 distance was evidently reduced, which was a consequence of the formation of the norbornene skeleton. Formally, the considered TS was evidently like transition states observed within the transition states of [3.3]-sigmatropic shifts [35]. It should be underlined that both TSs, as well as the intermediate **8**/BF_3_, exhibited polar natures (GEDT = 0.3–0.5 e). The further conversion of the TS1B/BF_3_ led directly to the valley of the target product. IRC calculations confirmed this.

## 3. Computational Details

The computational study was performed using the wb97xd/6-311+G(d) level of theory, and the Gaussian 09 package was the software [39]. The PlGrid infrastructure in the national computing centre “Cyfronet” was applied. A similar computational level has already been successfully used to explore mechanistic aspects of different-type cycloaddition processes, including Diels–Alder reactions [40,41,42,43]. All localised stationary points were characterised using vibrational analysis. It was found that starting molecules, as well as products, had positive Hessian matrices. On the other hand, all transition states (TS) showed only one negative eigenvalue in their Hessian matrices. Intrinsic reaction coordinate (IRC) calculations were performed for all optimised transition states. The presence of the solvent in the reaction environment (dichloromethane, nitromethane) was included using the IEFPCM (Integral Equation Formalism Polarisable Continuum Model) algorithm [44]. Calculations of all critical structures were performed for the temperature T = 298 K and pressure p = 1 atm. The results are collected in Table 2 and Table 3. Consistent with previous conventions in this paper, the pre-reaction complexes are denoted as MC, and the transition structures as TS.

The global electron density transfer (GEDT) [45] was calculated according to the formula:

GEDT = −ΣqA

where qA is the net charge, and the sum is taken over all the atoms of nitroalkene.

Global and local electronic properties of reactants were estimated according to the equations recommended earlier by Parr and Domingo [46,47]. In particular, the electronic chemical potentials (µ) and chemical hardness (η) were evaluated in terms of one-electron energies of FMO (E_HOMO_ and E_LUMO_) using the following equations:

μ ≈ (E_HOMO_ + E_LUMO_)/2  η ≈ E_LUMO_ − E_HOMO_

Next, the values of µ and η were then used for the calculation of global electrophilicity (ω) according to the formula:

ω = μ^2^/2η

Subsequently, global nucleophilicity (N) [48] can be expressed in terms of the equation:

N = E_HOMO_ − E_HOMO (tetracyanoethene)_

The local electrophilicity (ω_k_) condensed to atom k was calculated by projecting the index ω onto any reaction centre k in the molecule using *Parr* functions P^+^_k_ [49]:

ω_k_ = P^+^_k_·ω

The local nucleophilicity (N_k_) condensed to atom *k* was calculated using global nucleophilicity N and *Parr* functions P^−^_k_ [49] according to the formula:

N_k_ = P^−^_k_·N

The results are collected in Table 1.

## 4. Conclusions

Results of our wb97xd/6-311+G(d) (PCM) calculations shed light on the mechanistic aspects of cyclopentadiene with isopropyl 3-nitroprop-2-enate 2. It was found that, depending on the reaction conditions, the different mechanisms can be realised on the way, leading to target nitronorbornene molecular systems. In particular, in the DCM solution, two alternative reaction channels are possible and allowed from the kinetic point of view. Both reactions are controlled by the attack of the more electrophilic beta carbon centre at the nitrovinyl moiety of nitroalkene to the nucleophilically activated 1-position of the cyclopentadiene. In the first scenario, the adduct with the *exo*-orientation of the nitro group was formed, whereas, in the second one, the endo-nitro norbornene was formed. This conclusion correlates well with the experimental results. From the mechanistic point of view, considered processes should be classified as one-step but polar. The replacement of DCM with a more polar solvent created an additional way for the conversion of addends. This reaction channel leads to the zwitterionic intermediate characterised by “extended” conformation. Lastly, introducing the BF_3_ catalyst to the reaction environment substantially changed the mechanism on all considered paths. In particular, the cycloaddition leading to the *exo*-nitro norbornene was realised via the stage of the formation of a zwitterionic intermediate characterised by “cyclic” conformation. On the other hand, the competitive reaction path leading, finally, to the endo-nitro norbornene should be interpreted as the domino process, including the stage of the Hetero Diels–Alder reaction and the [3.3]-sigmatropic shift.

## Figures and Tables

**Table 1:** Global (global electrophilicity ω, global nucleophilicity N) and local electronic properties (local Parr functions P^+/−^_k_, local nucleophilicity N_k_, local electrophilicity ω_k_) of the cyclopentadiene **1** and isopropyl 3-nitroprop-2-enate **2**.

No.	Global Properties		Local Properties							
	ω [eV]	N [eV]	P^−^_C1_	P^−^_C2_	N_C1_ [eV]	N_C2_ [eV]	P^+^_C5_	P^+^_C6_	ω _C5_ [eV]	ω _C6_ [eV]
**1**	0.83	3.36	0.47	0.08	1.59	0.27				
**2**	2.31	1.36					0.005	0.414	0.01	0.95

**Table 2:** Energetic parameters for the reactions between cyclopentadiene 1 and isopropyl 3-nitroprop-2-enate **2** in light of the wb97xd/6-311+G(d) (PCM) calculations (ΔH, ΔG are in kcal/mol; ΔS is in cal/molK).

Solvent	Path	Transition	ΔH	ΔS	ΔG
DCM	**A**	**1** + **2** → **MCA**	−6.3	−40.2	5.7
		**1** + **2** → **TSA**	6.9	−51.0	22.1
		**1** + **2** → **3**	−32.6	−52.6	−16.9
	**B**	**1** + **2** → **MCB**	−6.7	−40.3	5.3
		**1** + **2** → **TSB**	5.9	−51.1	21.1
		**1** + **2** → **4**	−32.2	−50.4	−17.2
Nitromethane	**A**	**1** + **2** → **MCA**	−6.1	−39.8	5.7
		**1** + **2** → **TSA**	6.8	−50.8	21.9
		**1** + **2** → **3**	−32.4	−52.7	−16.7
	**B**	**1** + **2** → **MCB**	−6.5	−40.1	5.4
		**1** + **2** → **TSB**	5.7	−50.5	20.7
		**1** + **2** → **4**	−32.1	−50.4	−17.1
	**C**	**1** + **2** → **MCA**	−6.1	−39.8	5.7
		**1** + **2** → **TSC**	18.9	−47.6	33.1
		**1** + **2** → **7**	11.7	−46.4	25.6
DCM	**A**	**2** + BF_3_ → [**2**/BF_3_]	−6.1	−36.5	4.7
		**1** + [**2**/BF_3_] → **MCA/**BF_3_	−7.7	−41.2	4.6
		**1** + [**2**/BF_3_] → **TS1A/**BF_3_	−2.7	−50.6	12.4
		**1** + [**2**/BF_3_] → **5/**BF_3_	−13.3	−52.2	2.2
		**1** + [**2**/BF_3_] → **TS2A/**BF_3_	−11.0	−57.3	6.1
		**1** + **2**+BF_3_ → **3** + BF_3_	−32.6	−52.6	−16.9
	**B**	**1** + [**2**/BF_3_] → **MCB/**BF_3_	−8.5	−44.2	4.6
		**1** + [**2**/BF_3_] → **TS1B/**BF_3_	−5.3	−51.4	10.0
		**1** + [**2**/BF_3_] → **8/**BF_3_	−37.7	−55.1	−21.2
		**1** + [**2**/BF_3_] → **TS2A/**BF_3_	−14.8	−58.8	2.7
		**1** + **2** + BF_3_ → **4** + BF_3_	−32.2	−50.4	−17.2

**Table 3:** Key parameters of critical structures of reactions between cyclopentadiene **1** and isopropyl 3-nitroprop-2-enate **2** in light of the wb97xd/6-311+G(d) (PCM) calculations.

Solvent	Structure	Interatomic Distances [Å]						GEDT
		C1–C2	C2–C3	C3–C4	C4–C5	C5–C6	C6–C1	[e]
DCM	**1**	1.344	1.468	1.344				
	**2**					1.323		
	**MCA**	1.346	1.463	1.346	3.210	1.325	3.231	0.00
	**TSA**	1.398	1.398	1.389	2.336	1.388	2.166	0.32
	**3**	1.520	1.333	1.520	1.565	1.543	1.557	
	**MCB**	1.346	1.464	1.345	3.235	1.324	3.227	
	**TSB**	1.397	1.401	1.385	2.379	1.387	2.147	0.32
	**4**	1.515	1.334	1.517	1.565	1.538	1.579	0.00
Nitromethane	**1**	1.344	1.468	1.344				
	**2**					1.323		
	**MCA**	1.346	1.463	1.346	3.211	1.325	3.231	0.00
	**TSA**	1.399	1.398	1.388	2.364	1.388	2.150	0.33
	**3**	1.520	1.333	1.519	1.565	1.544	1.557	
	**MCB**	1.346	1.464	1.346	3.236	1.324	3.228	0.00
	**TSB**	1.398	1.402	1.383	2.421	1.388	2.127	0.34
	**4**	1.515	1.334	1.517	1.565	1.538	1.579	
	**MCA**							0.00
	**TSC**	1.415	1.411	1.367	4.371	1.416	1.855	0.61
	**7**	1.472	1.379	1.388	4.351	1.492	1.561	0.99
DCM	[**2**/BF_3_]					1.325		
	**MCA/**BF_3_	1.348	1.460	1.348	3.150	1.330	3.109	0.00
	**TS1A/**BF_3_	1.388	1.418	1.368	2.870	1.382	2.181	0.39
	**5/**BF_3_	1.481	1.372	1.392	3.398	1.489	1.579	0.93
	**TS2A/**BF_3_	1.483	1.361	1.417	2.347	1.488	1.606	0.61
	**MCB/**BF_3_	1.350	1.460	1.348	3.162	1.331	3.029	0.00
	**TS1B/**BF_3_	1.385	1.426	1.360	2.966	1.377	2.233	0.35
	**8/**BF_3_	1.546	1.491	1.328	3.688	1.488	1.548	0.34
	**TS2A/**BF_3_	1.482	1.369	1.406	2.379	1.484	1.606	0.47

## Data Availability

Not applicable.

## Sample Availability

Samples of the compounds are not available from the authors.
